# Lipid-Coated Mesoporous Silica Nanoparticles for the Delivery of the ML336 Antiviral to Inhibit Encephalitic Alphavirus Infection

**DOI:** 10.1038/s41598-018-32033-w

**Published:** 2018-09-18

**Authors:** Annette E. LaBauve, Torri E. Rinker, Achraf Noureddine, Rita E. Serda, Jane Y. Howe, Michael B. Sherman, Amy Rasley, C. Jeffery Brinker, Darryl Y. Sasaki, Oscar A. Negrete

**Affiliations:** 1 Department of Biotechnology and Bioengineering, Sandia National Laboratories, Livermore, CA USA; 2 Advanced Materials Laboratory, Sandia National Laboratories, Albuquerque, NM USA; 3 Chemical and Biological Engineering, University of New Mexico, Albuquerque, NM USA; 4 Center for Micro-Engineered Materials, Advanced Materials Laboratory, Albuquerque, NM USA; 5 Hitachi High Technologies America Inc., Clarksburg, MD USA; 6 Sealy Center for Structural Biology & Molecular Biophysics, University of Texas Medical Branch, Galveston, TX USA; 7 Biosciences and Biotechnology Division, Lawrence Livermore National Laboratory, Livermore, CA USA

## Abstract

Venezuelan equine encephalitis virus (VEEV) poses a major public health risk due to its amenability for use as a bioterrorism agent and its severe health consequences in humans. ML336 is a recently developed chemical inhibitor of VEEV, shown to effectively reduce VEEV infection *in vitro* and *in vivo*. However, its limited solubility and stability could hinder its clinical translation. To overcome these limitations, lipid-coated mesoporous silica nanoparticles (LC-MSNs) were employed. The large surface area of the MSN core promotes hydrophobic drug loading while the liposome coating retains the drug and enables enhanced circulation time and biocompatibility, providing an ideal ML336 delivery platform. LC-MSNs loaded 20 ± 3.4 μg ML336/mg LC-MSN and released 6.6 ± 1.3 μg/mg ML336 over 24 hours. ML336-loaded LC-MSNs significantly inhibited VEEV *in vitro* in a dose-dependent manner as compared to unloaded LC-MSNs controls. Moreover, cell-based studies suggested that additional release of ML336 occurs after endocytosis. *In vivo* safety studies were conducted in mice, and LC-MSNs were not toxic when dosed at 0.11 g LC-MSNs/kg/day for four days. ML336-loaded LC-MSNs showed significant reduction of brain viral titer in VEEV infected mice compared to PBS controls. Overall, these results highlight the utility of LC-MSNs as drug delivery vehicles to treat VEEV.

## Introduction

New World alphaviruses affect North, South, and Central America and pose a major public health threat as they are highly infectious and can result in fatal encephalitis in humans^1–3^. One of these alphaviruses, the Venezuelan equine encephalitis virus (VEEV), is classified as a Category B Agent by the CDC and NIAID due to ease of aerosolization of highly infectious virions and the lack of controlled vaccines and antivirals against the virus^3^. Because of its potentially debilitating health consequences, low infectious dose in humans, and stability in storage, VEEV is a potential bioterrorism agent and has been previously stockpiled in the US and USSR^2,3^. In addition to its use as a bioterrorism agent, natural VEEV outbreaks have resulted in equine and human infections in North and South America, causing high rates of fatality in equines (85%) and chronic neurological complications in humans^3–5^. The virus causes influenza-like symptoms in humans with 14% of infections resulting in neurological complications and sequelae, including disorientation, ataxia, depression, and convulsions^2,5^. With one percent of human infections resulting in mortality^4,5^, the development of new strategies to inhibit VEEV infection is critical to minimizing fatalities and complications of infection from both bioterrorism and natural outbreaks.

Several small molecule drugs have been developed that inhibit VEEV, but many are limited by high toxicity or low efficacy^6–11^. Recently, a highly effective small molecule inhibitor of VEEV was developed with the assistance of a high throughput, cell-based screen^4,6^. Referred to as ML336, this molecule was found to have a EC_90_ of 170 nM against a VEEV vaccine strain (TC-83) and reduce viral titer by 630,000-fold at nanomolar concentrations. In addition, intraperitoneal administration of ML336 to mice infected with TC-83 resulted in a 71% survival rate as compared to the 14% survival rate observed in untreated mice. While the potency of this drug at nanomolar concentrations and *in vivo* study results are encouraging, ML336 has limited solubility (0.04 mg/mL in PBS, pH 7.4) and limited stability (reduction of 17% and 35% of drug in PBS and mouse plasma, respectively, after 3 hours) in aqueous solutions^4^, potentially reducing its efficacy. To improve drug solubility and stability, we investigated utilizing a mesoporous silica nanoparticle-based platform to deliver ML336 for VEEV inhibition both *in vitro* and *in vivo*.

Mesoporous silica nanoparticles (MSNs) have been used in drug delivery systems to improve drug stability and solubility, protect cargo, target specific tissues, and enhance drug circulation time and controlled release^12,13^. MSNs have narrow particle and pore size distributions and can be optimized for various drug delivery applications by tuning particle size, pore size, surface properties, and the porous structure^14,15^. Established methods enable formation of MSNs with tunable pore size, endowing MSNs with a large surface area for drug adsorption (600–1000 m^2^/g)^12–15^. This property is particularly advantageous for loading water insoluble or unstable drugs, as the large surface area acts as a reservoir for hydrophobic drug in aqueous solution and can improve drug efficacy *in vivo*^16,17^. In addition, MSNs are stable in non-aqueous solutions and permit loading of hydrophobic drugs in organic solvents, giving them a distinct advantage over polymeric or liposomal nanoparticle delivery systems^18^. While MSNs are a promising carrier for ML336, drug-loaded MSNs can have low colloidal stability and are subject to aggregation in physiological solutions, reducing circulation time and preventing desirable cell uptake^19,20^. In addition, premature release of cargo from MSNs can be problematic^21^. In order to overcome these challenges, we investigated the application of a lipid-based coating to the exterior of ML336-loaded MSNs.

MSNs coated with supported lipid bilayers (lipid-coated MSNs (LC-MSNs)) have been employed in drug and protein delivery applications to improve colloidal stability and subsequent circulation time, biocompatibility, cargo loading and release, and tissue-specific targeting^19,21–23^. Encapsulation of the MSN within a conformal supported lipid bilayer via liposome fusion can improve colloidal stability in physiological solutions^19,20^ and prevent cargo release prior to cell internalization or some other external trigger^21^. Furthermore, a lipid bilayer coating offers an additional surface that can be functionalized independently of the MSN surface for tissue-specific targeting^19,21,22,24^. Finally, the inherent instability and broad size distribution of liposomes is overcome by adhesion to the MSNs to form LC-MSNs^21,22,25^. Thus, LC-MSNs harness the advantages and overcome the obstacles associated with MSNs and liposomes in one versatile platform for small molecule delivery.

In this work, we highlight the use of LC-MSNs for ML336 delivery to inhibit VEEV. LC-MSN characterization revealed uniformly sized particles coated with a lipid bilayer that maintained colloidal stability. The delivery vehicle was able to load and release ML336 in a manner that inhibited virus *in vitro*. Cell internalization studies suggest a clathrin-mediated endocytosis pathway is involved in uptake of LC-MSNs. Finally, ML336 loaded LC-MSNs showed *in vivo* viral inhibition in a murine model of VEEV infection. Overall, this work demonstrates the first use of a nanoparticle-based system for the delivery of ML336. The successful inhibition of virus achieved with this platform could have widespread benefit in combatting VEEV and other viral infections resulting from bioterrorism or natural causes.

## Results and Discussion

### ML336 loaded LC-MSNs

**Figure 1:**
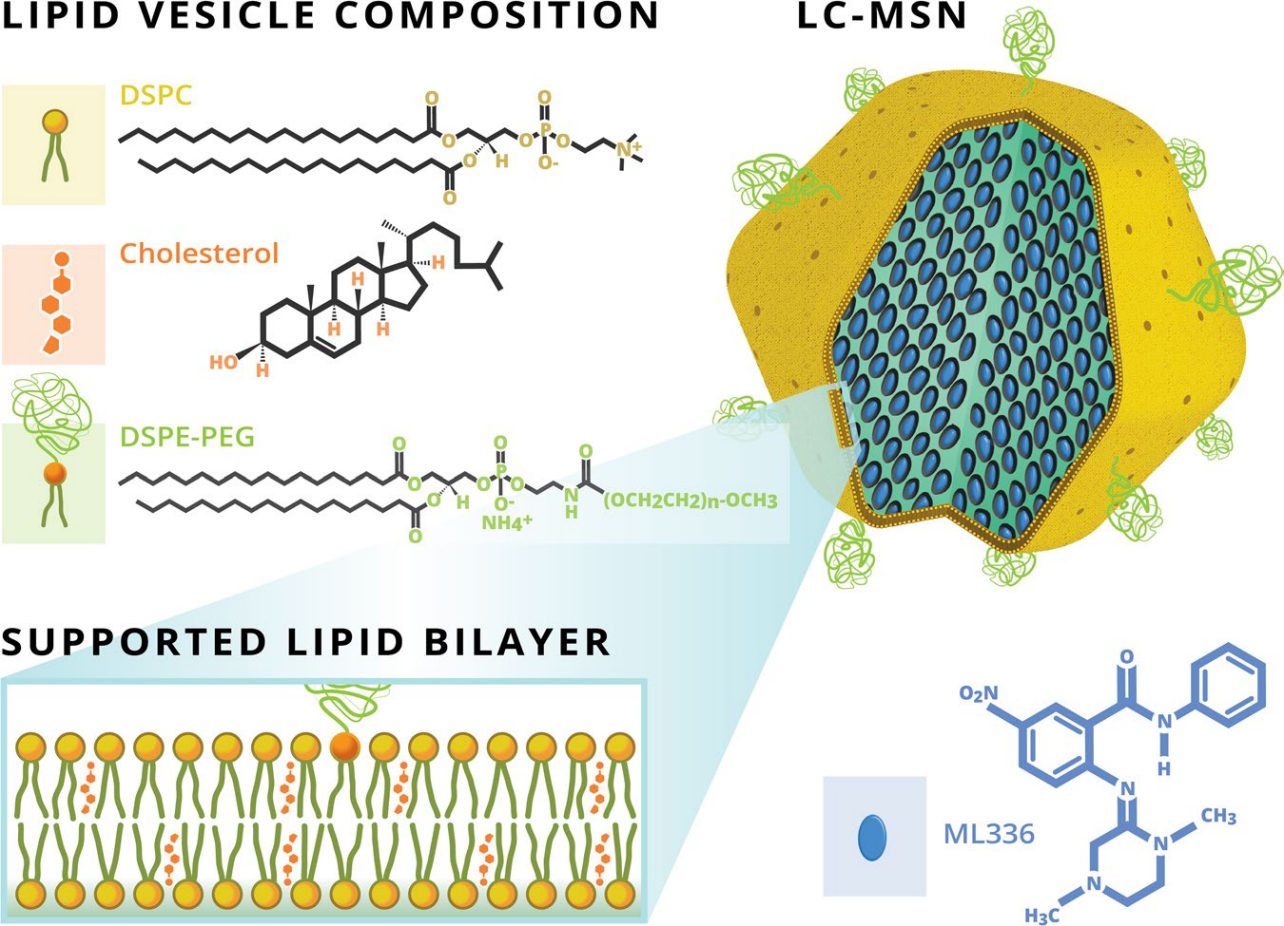
Schematic of ML336 loaded LC-MSNs. The antiviral ML336 was incubated with MSNs prior to vesicle fusion with liposomes containing a composition of 77.5% DSPC:2.5% DSPE-PEG2000:20% cholesterol at mole ratios.

The small molecule ML336 was recently discovered to have antiviral drug properties against VEEV^4^. While proven effective both *in vivo* and *in vitro*, ML336 has limited solubility in aqueous solution, necessitating a delivery vehicle to improve drug stability and enable controlled release. In this study, a hybrid liposome-mesoporous silica nanoparticle technology was utilized that takes advantage of the loading capabilities and uniform size of MSNs and the biocompatibility and retention capabilities of liposomes in one drug delivery platform (Fig. 1)^19,22,26,27^. Referred to as LC-MSNs (often referred to as “protocells” in previous work^22^), these particles have the potential to protect and control the release of ML336 as well as be modified for tissue-specific targeting in future iterations of the LC-MSN technology^19,20^.

**Figure 2:**
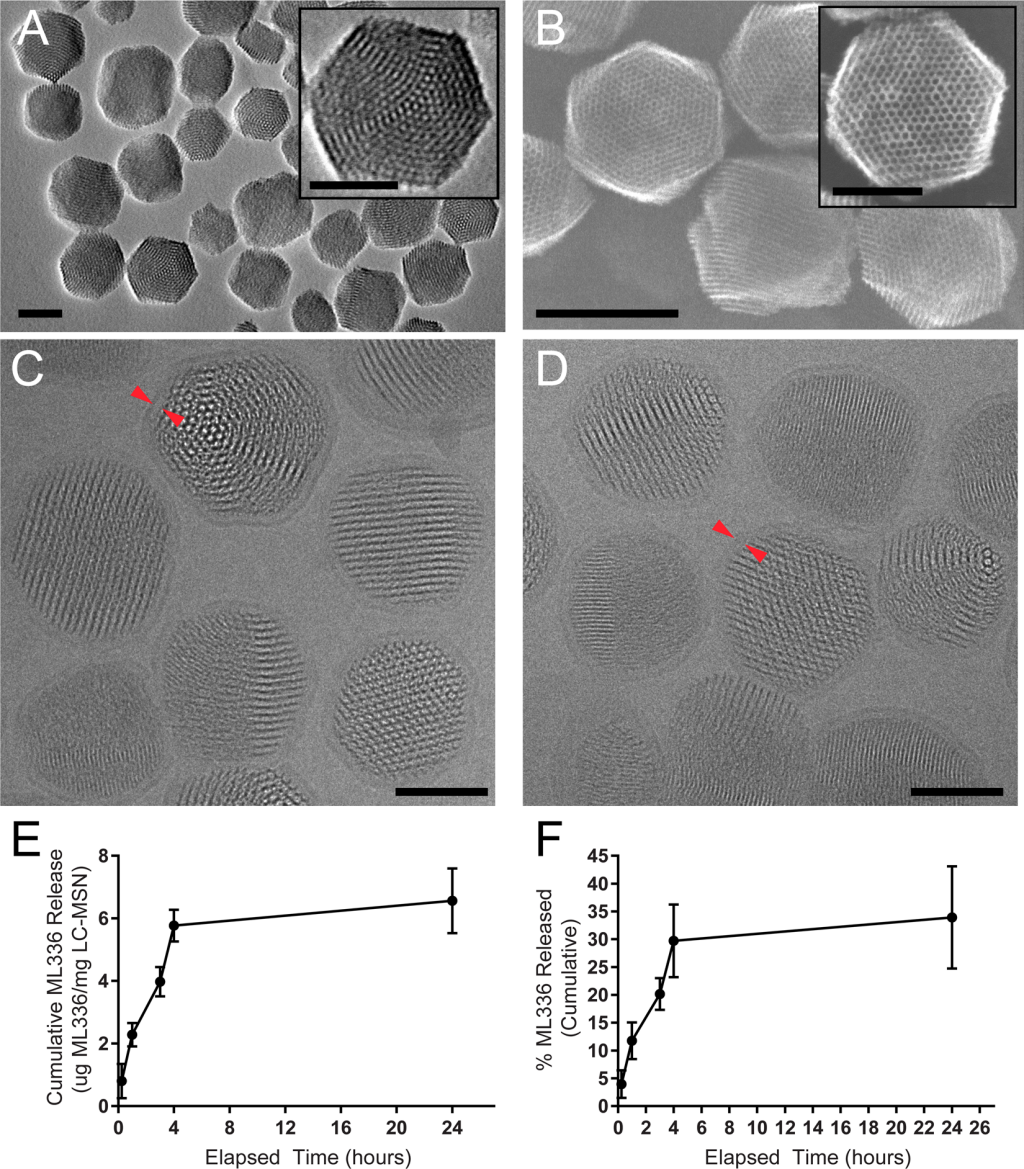
Characterization of ML336 LC-MSNs. TEM (**A**), SEM (**B**) images of MSNs and cryo-EM images of (**C**) ML336-loaded and (**D**) unloaded LC-MSNs (all scale bars = 50 nm except (**B**) bottom left scale bar = 100 nm). Red arrows point out examples of the lipid bilayer. (**E**) Cumulative and  (**F**) percent release (normalized to total ML336 loaded) of ML336 from LC-MSNs. Data represent mean ± standard deviation, n = 6.

LC-MSN formation was modified based on past methods^19,20^. First, monosized sub-150 nm MSNs were produced by up-scaling previous synthesis protocols. Optimized large batch synthesis procedures yielded highly homogeneous nanoparticles without alteration in structure or size. As shown by transmission electron microscopy (TEM) (Fig. 2A), the nanoparticles of approximately 75 nm were narrow in size distribution and displayed a hexagonal porous structure (Fig. 2A, inset). Their homogeneous colloidal size was also confirmed by DLS (95.9 ± 2.1 nm, PDI = 0.07 ± 0.01) (Table 1). Additionally, scanning electron microscopy (SEM) analysis (Fig. 2B) was performed in order to highlight the 3D hexagonal shape of the MSNs and, importantly, the open porous structure. As demonstrated in Fig. 2B (insert) and Fig. S1, surface accessible pores were clearly observed. In addition to SEM observation, N_2_ sorption also provided evidence on the pore shape and its surface accessibility. The resulting isotherm (Fig. S1C) showed a steep increase in adsorption characteristic of a capillary condensation in mesopores capillary evaporation on the desorption branch, supporting the presence of uniform cylindrical mesopore open at both ends (surface-accessible). Accessibility of the pores was indirectly confirmed by the high (BET) accessible surface area found for these MSNs. Furthermore, 100 pore diameters were measured on the SEM micrograph and their average was found to be 2.65 ± 0.29 nm which is in the same order of magnitude of the average pore size found by N_2_ sorption using DFT theory (~3.5 nm) (Fig. S1).

**Table 1:** Z-average diameter, PDI, and zeta potential for MSNs, liposomes, unloaded LC-MSNs, loaded MSNs, and loaded LC-MSNs.

Particle Type	Medium	Z-average Diameter (nm)	PDI	Zeta Potential (mV)
MSN	Water	95.9 ± 2.1	0.072 ± 0.01	−25.0 ± 0.42
Liposome	50:50 PBS:Water	125.03 ± 2.1	0.090 ± 0.01	−0.78 ± 0.20
Unloaded LC-MSN	PBS	149.5 ± 1.5	0.116 ± 0.01	−0.263 ± 0.41
Loaded MSN	PBS	487.4 ± 108	0.351 ± 0.06	NA
Loaded LC-MSN	PBS	164 ± 1.5	0.144 ± 0.02	−1.76 ± 0.26

Liposomes were composed of 77.5:20:2.5 DSPC:Cholestrol:DSPE-PEG(2000), which was chosen to ensure formation of a stable bilayer and to enhance colloidal stability of the resulting LC-MSNs. The primary lipid component, DSPC, was selected due to its saturated acyl chain, as previous work has indicated that unsaturated lipids may contribute to reduced colloidal stability of LC-MSNs over time^19^. Cholesterol was used to improve control over bilayer fluidity and leakage^22,28^ and a PEGylated DSPE was included to increase circulation time and reduce protein adsorption to the LC-MSN surface^19,22,26,27^. To assemble LC-MSNs, liposomes were applied to MSNs under sonication at a 5:1 liposomes:MSN mass ratio, a similar ratio to those used previously (2:1–4:1) to produce high quality LC-MSNs^19^. Fusion between the negatively charged MSN and the zwitterionic liposome occurs due to Van der Waals and electrostatic interactions and the lipophilic nature of the MSNs^15,19,29,30^.

To ensure successful formation of LC-MSNs, particles were evaluated for size, charge, and morphology. In previous studies, increased particle size upon addition of a lipid bilayer to MSNs has been observed and is indicative of successful bilayer formation^19,21,28,31,32^. Results were similar in this work, as application of the lipid bilayer increased MSN particle diameter from ~96 nm to ~150 nm and 164 nm for unloaded and ML336-loaded particles, respectively (based on DLS measurements), while maintaining a low PDI and thus good size homogeneity (Table 1). A neutralized surface charge of particles is another measure of successful MSN-liposome fusion^31,33^, as a reduction of MSN surface charge is observed due to charge shielding of negatively charged deprotonated silanols on the MSN surface by the zwitterionic lipid bilayer^19^. Here, uncoated MSNs had a zeta potential of −25.0 ± 0.42 mV, similar to what has been reported in literature previously^13,19,20^, which was reduced to nearly neutral when coated with a lipid bilayer (−0.263 ± 0.41 mV, Table 1). As a final confirmation of successful bilayer application, a uniform lipid bilayer was observed in cryo-EM images (Fig. 2C,D). Analysis of the cryo-EM images indicated a LC-MSN diameter of 88.1 ± 11.8 nm and 86.5 ± 12.0 nm and a bilayer thickness of 6.0 ± 0.94 nm and 5.4 ± 0.91 nm for loaded and unloaded LC-MSNs, respectively. The smaller diameter of the LC-MSNs determined by cryo-EM compared to DLS is consistent with previous reports^19^. The larger diameter observed in loaded LC-MSNs via DLS size analysis (Table 1) suggests the surface adsorbed ML336 may affect the hydrodynamic radius of the particle due to changes in surface hydrophobicity. Overall, ML336-loaded LC-MSNs were successfully fabricated.

While MSNs offer many favorable features for small molecule delivery^12–15^, aggregation of MSNs without surface modification or external coatings is a common occurrence in high ionic strength physiologically relevant media due to a reduction in the Debye length and correspondingly the degree of electrostatic repulsion^12,17,20^. As might be predicted, ML336 loaded MSNs that were not coated with a lipid bilayer aggregated immediately in PBS (Table 1, Fig. S2A). In contrast, loaded LC-MSNs maintained colloidal stability for at least four days (Fig. S2A), indicating their utility for both *in vitro* and *in vivo* applications. Taken as a whole, zeta-potential, cryo-EM and stability studies indicate the formation of a complete, conformal and uniform lipid bilayer on ML336-loaded LC-MSNs.

ML336 loading in LC-MSNs was determined to be about 20 µg ML336/mg LC-MSN, as measured by subtracting the amount of ML336 lost in the post-lipid-coating and loading washes from the total mass of ML336 loaded (Fig. S2B–D). A linear burst release of ML336 was observed to occur in the first 4 hours, with little additional release thereafter (Fig. 2E,F). Overall, LC-MSNs released about 6.6 µg ML336/mg LC-MSNs in 24 hours, which correlated to 34% release of the loaded ML336 (Fig. 2E,F; Table 2). No additional release was observed after 4 additional days. Similar release was observed when LC-MSNs were incubated in PBS at pH 5, which mimics the intracellular endosome. The ML336 release observed here was similar to small molecule release from lipid coated MSNs in previous studies, where 0–35% release of loaded cargo was observed in ~10 h at pH 7 for several different lipid bilayer compositions^19,21,31–33^. When the pH was dropped to 5, no additional release was observed, confirming what has been observed for a similar lipid bilayer composition previously^19^. In other reports where additional and sometimes nearly complete release of cargo from lipid-coated MSNs has been reported at low pH^32,33^, specific acid-sensitive lipids have been employed to promote cargo release under acidic conditions. While still under investigation, the technology presented here could be modified to be acid-sensitive by adjusting the lipid composition of the lipid bilayer. However, the limited release at low pH observed in these studies could be beneficial, as it minimizes premature release and degradation of cargo in the endosomal compartment.

**Table 2:** Summary of ML336 Release from LC-MSNs.

Total ML336 Loaded (μg ML336/mg LC-MSN)	Total ML336 Released (μg ML336/mg LC-MSN)	% ML336 Released
20 ± 3.4	6.6 ± 1.3	33.5 ± 6.6

Complete ML336 release was observed when LC-MSNs were incubated in methanol, which is expected to disrupt the lipid bilayer and effectively extract ML336 from the MSN (Fig. S2E,F). This result suggests that the limited solubility of ML336 in aqueous solution prevents its release from the MSN core even after the expected disruption of the lipid bilayer at pH 5^33^. Previously, MSNs loaded with hydrophobic drugs have shown reduced solvation, possibly retarding or preventing drug release prior to self-erosion of the silica matrix^34^. As degradation of the silica matrix is highly dependent upon surface functionalization, loaded cargo, relative concentration of particles, and the surrounding environment^34,35^, cargo release from LC-MSNs will be dependent upon specific conditions in both *in vitro* and *in vivo* environments.

To enhance loading and release in future iterations of this technology, the MSN surface could be modified to optimize interactions between the MSN and ML336^14,15,36^. The hydrophobicity of ML336 requires loading in a non-polar solvent (DMSO was used in these studies), while release occurs in physiological conditions (buffered aqueous solutions). As different properties dictate the interactions between ML336 and MSNs in aqueous vs. non-aqueous solvents, it may be possible to maximize MSN-ML336 interactions in DMSO to enhance loading while minimizing MSN-ML336 interactions in PBS (or other aqueous solutions) to enhance release^36^. Overall, the results presented here indicate successful loading and release of ML336 from uniform LC-MSNs of high colloidal stability, providing an excellent prototype for future optimization and additional analysis in both *in vitro* and *in vivo* studies.

### ML336 loaded LC-MSN viral inhibition *in vitro*

To evaluate the performance of ML336-loaded LC-MSNs *in vitro*, their ability to inhibit virus in infected HeLa cells was assessed. First, a baseline was determined using soluble ML336, which inhibited TC-83 virus in a dose-dependent manner on HeLa cells with an IC-50 of 163 nM at 24 h (Fig. S3A,B). The wildtype VEEV, a BSL-3 agent, was inhibited by ML336 in a similar manner (Fig. S3C,D) but was not used in subsequent studies due to associated high risk. The inhibition of VEEV by ML336 observed here was similar to previous studies^4^.

**Figure 3:**
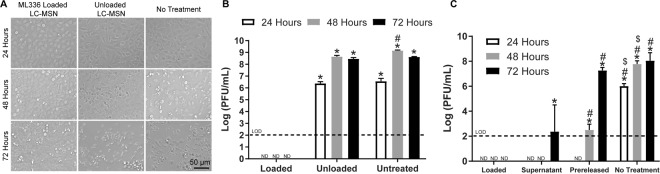
ML336 loaded LC-MSNs inhibited virus *in vitro*. (**A**) Phase microscopy images of cells 24, 48, and 72 hours post infection. Noticeable cell death is observed in the unloaded LC-MSN and no treatment groups as compared to loaded LC-MSN treatment group. (**B**) PFU/mL for loaded, unloaded, and untreated groups (*significantly different than loaded group at same timepoint, #significantly different than unloaded group at the same timepoint; n = 3 technical replicates and 3 biological replicates) (**C**) PFU/mL for loaded, supernatant, pre-released, and untreated groups. (*significantly different than loaded group at same timepoint, ^#^significantly different than supernatant group at the same timepoint, ^$^significantly different than pre-released group at the same timepoint; p < 0.05; data is depicted as mean ± standard deviation, n = 3 technical replicates and 5 biological replicates).

Cytotoxicity of ML336-loaded and unloaded LC-MSNs was assessed with HeLa cells. No visible differences in viability at 48 h was observed via LIVE/DEAD staining (Fig. S4), in line with the high biocompatibility observed in cells treated with LC-MSNs previously^19,33,37^ and with the limited toxicity observed when cells are treated with MSNs at a concentration less than 100 µg/mL^13^. To determine if ML336-loaded LC-MSNs inhibited virus, HeLa cells infected with the TC-83 virus were treated with ML336-loaded and unloaded LC-MSNs. Similar to soluble ML336, ML336-loaded LC-MSNs also inhibited virus in a dose-dependent manner, indicating that total ML336 release is proportional to LC-MSN mass and providing a method to tune drug dosage in a facile manner (Fig. S5). ML336 loaded LC-MSNs significantly decreased viral load by at least 4 orders of magnitude after 24 h and 6 orders of magnitude after 48 h and 72 h (Fig. 3A,B), a greater reduction than previously observed for other small molecule VEEV inhibitors^9,11^ and similar to what has been observed for soluble ML336^4^. Overall, these results indicate that ML336 loaded LC-MSNs can successfully inhibit VEEV.

As discussed above, little to no additional ML336 release from LC-MSNs incubated in PBS was observed after four hours (Fig. 2E,F). However, release of hydrophobic ML336 could depend heavily on the local microenvironment, especially if partially controlled by silica degradation^34,35^. In order to evaluate if LC-MSNs were effective after the initial 4 h burst release *in vitro*, ML336 loaded LC-MSNs were incubated in Opti-MEM for 4 h, called “Pre-released LC-MSNs”, and then separated from the supernatant. TC-83 infected HeLa cells were then treated with LC-MSN supernatant and pre-released LC-MSNs and compared to cells treated with loaded LC-MSNs and untreated cell controls. LC-MSN supernatant inhibited virus at a similar level to ML336 loaded LC-MSNs until the 72 h timepoint, at which point loaded LC-MSNs inhibited virus to a greater extent (Figs 3C and S6). This indicates that while released ML336 remains bioactive, the LC-MSNs protect ML336 over time and continually release additional ML336 in a manner different from our test tube release studies. While pre-released LC-MSNs inhibited virus in a similar manner to loaded LC-MSNs and LC-MSN supernatant at 24 h, by 48 h the extent of viral inhibition was significantly lower than cells treated with loaded LC-MSNs or LC-MSN supernatant. By 72 h, pre-released LC-MSNs showed no additional inhibition as compared to cells with no treatment (Figs 3C and S6). This indicates that LC-MSNs release additional ML336 after the initial four hour burst release, which may either be undetectable in the loading and release studies or does not occur prior to cell internalization and disruption of the lipid bilayer^33^. Taken as a whole, this data suggests that release from LC-MSNs occurs for longer than four hours (possibly up to 48 hours) and may depend on intracellular uptake. In addition, these studies were reproducible across multiple studies that employed different batches of particles (Fig. S6), indicating the robustness of the technology as a whole.

### LC-MSN cellular entry mechanism

**Figure 4:**
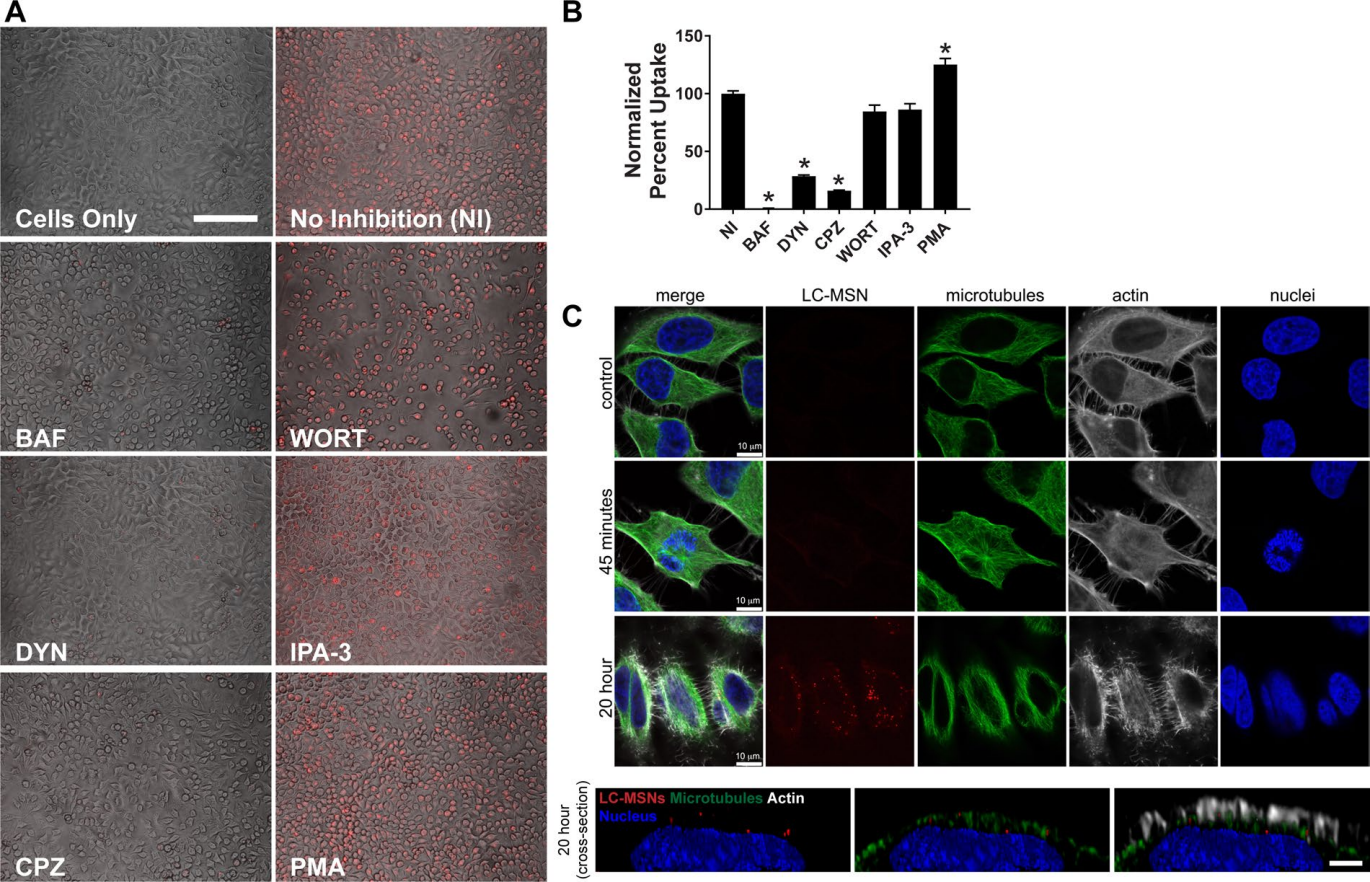
LC-MSN cellular internalization by clathrin-mediated endocytosis. (**A**) LC-MSNs containing a Cy3 dye label were added to inhibitor treated HeLa cells and the uptake efficiency was visualized using bright-field and fluorescent image overlays, or (**B**) quantified through flow cytometry. The inhibitor panel included those targeting pH dependent endocytosis (BAF), clathrin-mediated endocytosis (CPZ, DYN), macropinocytosis (WORT, IPA-3) and caveolae-mediated endocytosis (PMA, DYN), while untreated cells with (NI) and without LC-MSN (cells) addition served as controls. (**C**) HeLa cells treated with Cy3 labeled LC-MSNs for 45 min or 20 h were fixed and stained for microtubules with anti a-tubulin antibodies, actin with phalloidin, and nuclei with DAPI. Confocal images were acquired and 3D cell images were subjected to isosurface rendering to reveal time-dependent internalization of LC-MSNs. Scale bars for part A = 25 μm. Scale bars for part C, top panel = 10 μm; bottom panel = 2 μm. (*Significantly different than no inhibition group; p < 0.05; data is depicted as mean ± standard deviation, n = 3 technical replicates).

To begin to understand the dependency of LC-MSN cellular internalization on lipid bilayer disruption and complete drug cargo release, we first investigated whether LC-MSNs enter cells through endocytosis. LC-MSNs conjugated with affinity ligands are known to enter cells using trafficking pathways of the targeting receptor. For example, cholera toxin B conjugated LC-MSNs use lipid raft endocytosis for internalization after binding the GM1 ganglioside^20^. LC-MSNs have also been formulated to avoid non-specific uptake in blood circulation^19^. However, LC-MSN uptake in static conditions represented in these studies is not well understood. To determine whether LC-MSNs undergo cellular internalization through endocytosis, fluorescent LC-MSNs containing Cy3-labeled MSN cores were used to facilitate visualization and quantitation of entry into HeLa cells while in the presence of various endocytosis inhibitors. HeLa cells were treated with a pH dependent endocytosis inhibitor (BAF), clathrin-mediated endocytosis inhibitors (CPZ, DYN), caveolae-medicated endocytosis inhibitors (PMA, DYN) or macropinocytosis inhibitors (wort and IPA-3), for 1 h prior to the addition of Cy3-labeled LC-MSNs. Cells were vigorously washed to remove free particles and then examined by microscopy methods. Cy3-labeled LC-MSNs were readily internalized by HeLa cells under untreated (no inhibitor (NI)) conditions and in the presence of wort, IPA-3, and PMA. On the other hand, in the presence of BAF, CPZ and DYN the cellular uptake of LC-MSNs was clearly inhibited suggesting the role of clathrin-mediated endocytosis in cellular internalization of LC-MSNs (Fig. 4A).

To quantify the results obtained by microscopy, flow cytometry was used to measure the percentage of internalized Cy3-labeled LC-MSNs. HeLa cells were treated with the panel of endocytosis inhibitors for 1 h prior to and during incubation with fluorescent LC-MSNs. Before flow cytometry analysis, the cells were washed to remove unbound particles. Again, an inhibitor of endosomal acidification (BAF) almost completely inhibited LC-MSN internalization, and those of clathrin-mediated endocytosis dramatically reduced LC-MSN uptake by 71% with DYN and 84% with CPZ (Figs 4B and S7A). As viruses commonly use endocytosis for cellular entry, we confirmed the specificity of these inhibitors with viruses known to enter HeLa cells via endocytosis using vesicular stomatitis virus for clathrin-mediated endocytosis or Rift Valley fever virus for caveolae-mediated endocytosis dependent entry (Fig. S7B,C)^38^.

To confirm the labeled LC-MSNs were internalized and not on the cell surface, high resolution confocal microscopy techniques were employed. HeLa cells were incubated with LC-MSNs for either 45 min or 20 h, washed and then fixed for immunofluorescence staining. HeLa cells incubated with LC-MSNs for 20 hours were internalized as indicated by 3D rendering of LC-MSNs with actin, microtubules and nuclei intracellular markers (Fig. 4C). An actin stain was used to mark the periphery of the cell as actin filaments are concentrated at the cell periphery and form a 3D network that determines cell shape. Microtubule labeling using tubulin antibodies provided another reference for intracellular localization and depth of LC-MSNs within the cell. The LC-MSNs were found beneath the actin filaments, on the same plane as the microtubules, and above the cell nucleus. Further, these data indicated a time-dependent mechanism of entry as particles were not seen intracellularly at 45 min (Fig. 4C). Identifying the LC-MSNs cell entry pathway as clathrin-mediated endocytosis may provide a mechanism to design additional drug/cargo release at the site of LC-MSN accumulation. Overall, these results motivated a further investigation of the ability of ML336 loaded LC-MSNs to inhibit virus *in vivo*.

### ML336 loaded LC-MSN viral inhibition *in vivo*

**Figure 5:**
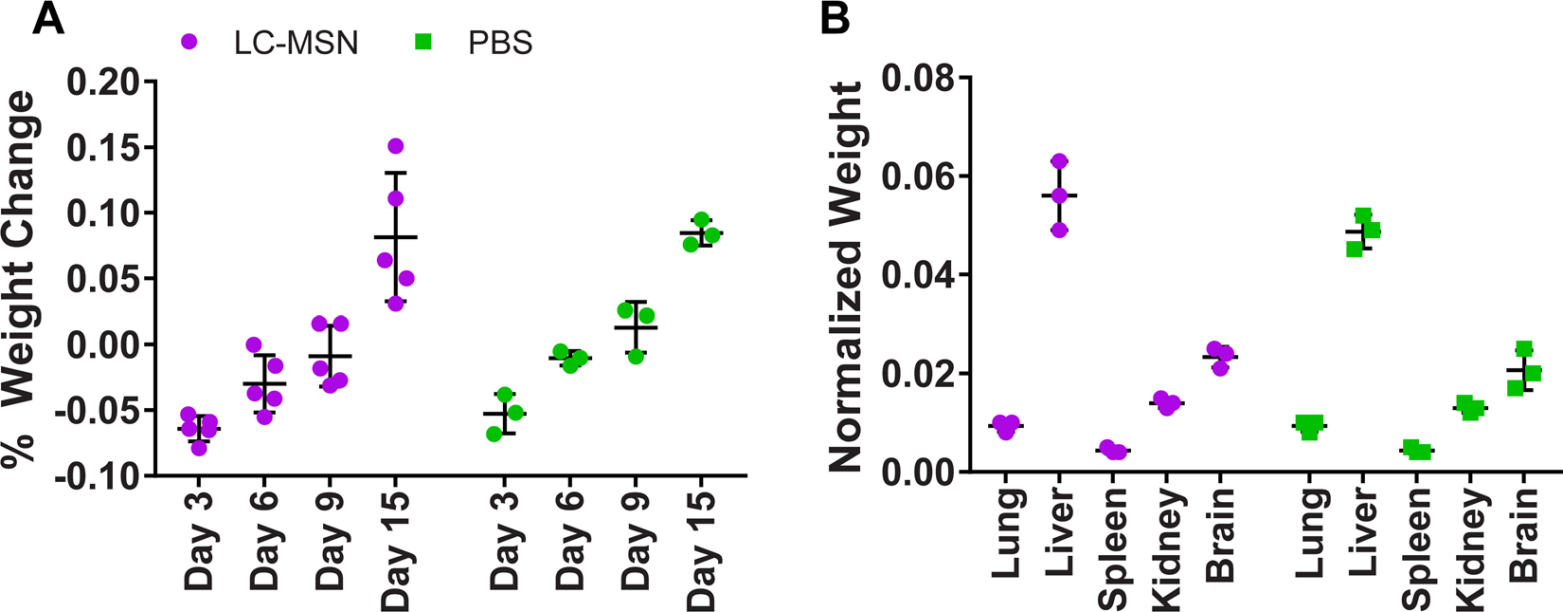
Unloaded LC-MSNs do not affect animal weight in safety studies. (**A**) Percent weight change in animals dosed with unloaded LC-MSNs or PBS alone over the course of 15 days. (**B**) Normalized weights of lung, liver, spleen, kidney, and brain to total animal weight in animals dosed with unloaded LC-MSNs or PBS alone over the course of 15 days (data is depicted as mean ± standard deviation).

As with all nanoparticle-based systems, the potential for LC-MSNs to dissolve, aggregate, and interact with living cells and animal tissues is dependent upon properties specific to their unique composition^14^. In addition, the toxicity of MSNs and LC-MSNs in general has yet to be fully assessed and can vary depending upon size and surface properties^12,13,18^. Thus, prior to conducting animal studies to evaluate antiviral efficacy, a safety study was conducted to determine if the LC-MSNs developed in this work affected mouse weight and survival over fifteen days. Mice were injected with 1 mg unloaded LC-MSNs twice daily for four days, and all mice survived treatment with no significant differences in total animal weight between LC-MSN and PBS treated groups (Fig. 5A). Previously, MSNs have been seen to accumulate in the spleen, liver, bladder, and kidneys^13,14^. In this work, no significant differences were observed between lung, liver, spleen, kidney, or brain weights in mice treated with LC-MSNs as compared to PBS only (Fig. 5B). A lack of weight change in tissue where bioaccumulation was likely the highest (liver, spleen and kidney) further suggests a lack of LC-MSN toxicity. Similarly, tissues processed for histology revealed a normal morphology in brain, spleen and kidney sections in LC-MSN treated mice while only very mild changes of some sections were seen in livers and lungs of nanoparticle dosed animals (Fig. S8)

In summary, we observed no overt toxicity in mice when a 0.11 g LC-MSNs/kg mouse dose was administered each day for four days, resulting in a total possible accumulated mass of silica nanoparticles of 0.44 g/kg. This correlates well with previous work, where MSN toxicity in mice was observed to be problematic when MSN were administered one time at 1.2 g/kg by IV injection^39^ but little to no toxicity was observed when 0.2 g/kg was administered once a day for 10 days^40^. The nanoparticles used in the work presented here also include the addition of the lipid bilayer, likely improving biocompatibility, increasing circulation time, and reducing toxicity as compared to uncoated MSNs^12^. Overall, results indicated that administration of LC-MSNs did not cause significant toxicity in mice, motivating further studies to investigate the ability of drug-loaded particles to inhibit viral infection.

**Figure 6:**
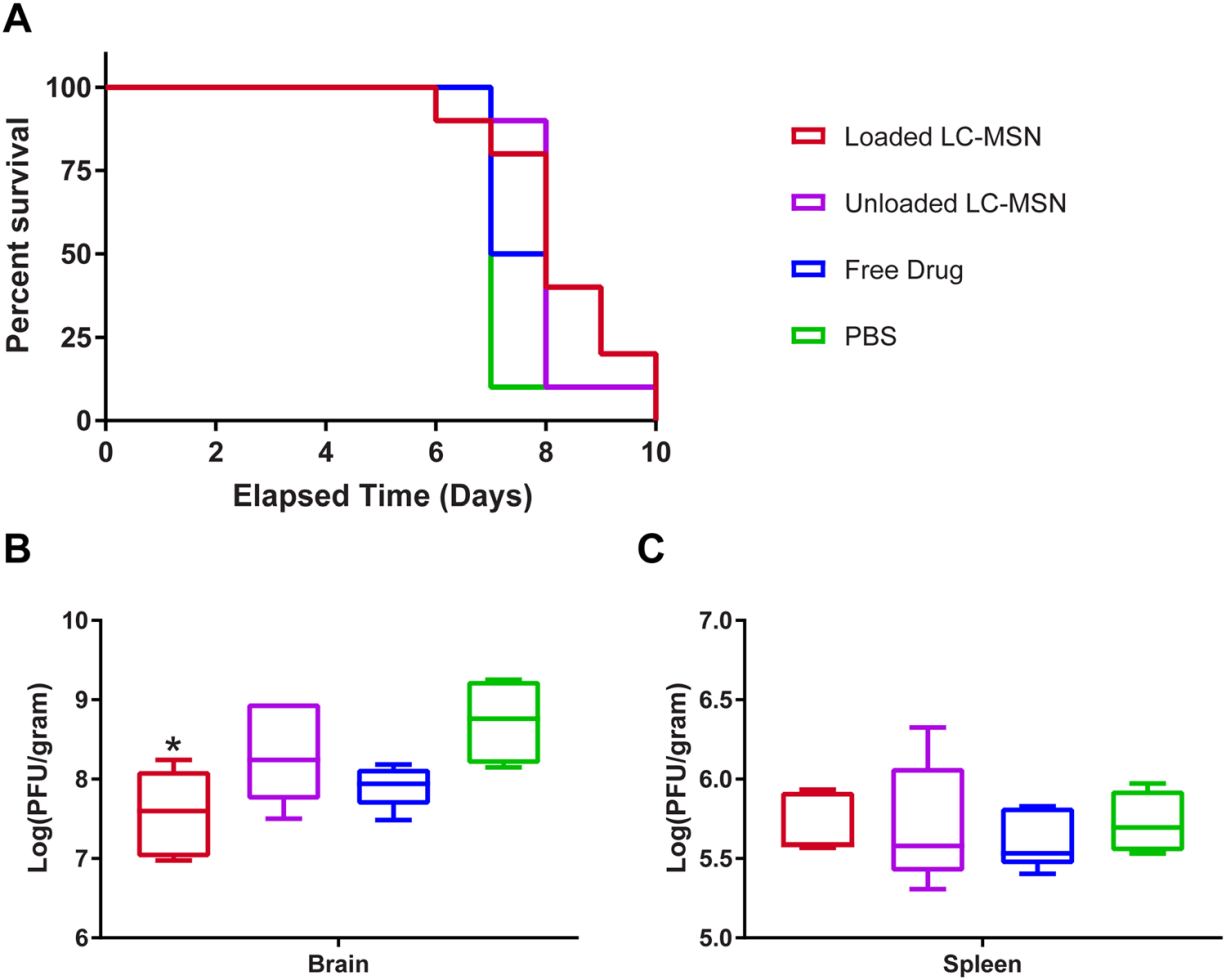
ML336 loaded LC-MSNs show reduction of brain infection *in vivo*. (**A**) Survival curve for TC-83 infected mice treated with 1.0 mg ML336 loaded LC-MSNs twice a day for 4 days. (**B**) Viral load in brain and (**C**) spleen normalized to organ mass after 4 days of infection and treatment with 1.5 mg ML336 loaded LC-MSNs (*significantly different from PBS group; p < 0.05; data is depicted as mean ± standard deviation).

In the first set of animal studies, mice were treated with 1 mg loaded LC-MSNs, unloaded LC-MSNs, free ML336 or PBS only. Mice treated with ML336 loaded LC-MSNs showed greater survival than mice in the other three groups, though this result was not statistically significant (Fig. 6A). As we observed that LC-MSNs inhibited virus in a dose-dependent manner in *in vitro* studies (Fig. S5), we were interested to see if an increased LC-MSN dose would improve animal outcomes. Thus, in the second set of animal studies, mice were dosed with 1.5 mg LC-MSNs. No differences were observed in spleen viral load (Fig. 6C) and very limited viral loads were detected in livers, kidneys, or serum (Fig. S9), similar to what has been observed in past studies characterizing TC-83 intranasal infection in C3H/HeN mice^8^. However, viral load in the brain was significantly reduced by about 10-fold in the ML336 loaded LC-MSN treated mice as compared to the PBS treated mice after 4 days (Fig. 6B). As viral load in mice treated with free ML336 was not significantly different from PBS treated mice, LC-MSNs may protect ML336 or increase circulation time in a manner that permits enhanced antiviral activity. In the future, a larger number of animals will help further elucidate trends. Overall, these results are encouraging and indicate the potential utility of ML336-loaded LC-MSNs in inhibiting VEEV infection.

No FDA approved therapeutics are available for VEEV^3,4^, though several studies have highlighted the ability of small molecules to inhibit VEEV both *in vitro* and *in vivo*^6–11^. Several small molecule inhibitors of VEEV have shown moderate success, though drug toxicity has remained an issue^7,10^. In addition, similar to the results in this study (Fig. 5B), animals treated with small molecule VEEV inhibitors show less than 10-fold brain viral titer reduction as compared to untreated control groups^7,11^, which may result in increased neurological impairment. Future iterations of the LC-MSNs presented in this work have the potential to improve these outcomes by reducing toxicity and enabling targeting specific to the blood-brain barrier in the case of VEEV infections. First, LC-MSNs can prevent toxicity by (1) reducing the concentration required for drug efficacy, both through improvements in drug solubility/stability as well as circulation time^12,19^, and (2) protecting the cellular microenvironment from harmful cargo prior to triggered release, either through rupture of the lipid bilayer or a specific chemically triggered mechanism^22^. Second, the LC-MSN lipid bilayer can be modified to specifically target a tissue of interest, such as the blood-brain barrier^20,22^. LC-MSNs are particularly advantageous because properties of the lipid bilayer and the MSN can be independently tuned, enabling simultaneous optimization of the lipid bilayer for tissue-specific targeting and the core to maximize drug-specific loading. Overall, the studies presented here highlight the ability of drug loaded LC-MSNs to prevent viral infection in one particular case, but the versatility and modifiability of this technology will enable use of LC-MSNs to prevent viral infection in a variety of future applications.

## Conclusion

In this work, we presented the first use of LC-MSNs to deliver ML336 for TC-83 VEEV inhibition both *in vitro* and *in vivo*. ML336 loaded LC-MSNs were successfully coated with a lipid bilayer, which significantly improved colloidal stability, and enabled sustained release of cargo over the course of 4 hours. Viral loads were reduced by 4–6 orders of magnitude in TC-83 VEEV infected HeLa cells treated with ML336 loaded LC-MSNs, which was repeatable across several particle batches in different studies. Furthermore, *in vitro* studies indicated the possibility of additional release of ML336 after cellular internalization via clathrin-mediated endocytosis and enhanced ML336 stability when loaded in LC-MSNs. Safety studies indicated that LC-MSNs were not toxic in mice at the doses administered in this study. In mice infected with TC-83 VEEV, treatment with ML336-loaded LC-MSNs resulted in a significant reduction of viral load in the brain after four days of treatment. Overall, these studies highlight the utility of LC-MSNs for drug delivery in antiviral applications, and provide an additional defense against VEEV and other alphaviruses in the cases of natural infection or bioterrorism.

## Methods

### MSN fabrication and characterization

Both small and large batch syntheses of monosized hexagonally-structured MSNs were prepared as previously described^19,41,42^, with modifications. An up-scaled batch of hexagonal small pore particles was prepared in a 1 L beaker in which cetyl trimethylammonium bromide (CTAB, 1.45 g) was dissolved in 750 mL of aqueous ammonium hydroxide (0.32 M), and then placed sealed in a heated silicone oil bath (50 °C, 2 h) stirring at high speed (650 rpm). A tetraethyl orthosilcate (TEOS) (Sigma) solution was prepared at 0.88 M in ethanol (15 mL) and subsequently combined with the CTAB surfactant solution. The reaction was stirred vigorously for 1 h unsealed and then aged overnight (~18 h) in a 50 °C silicone oil bath without stirring. The remaining volume was transferred to a 500 mL glass bottle and capped for an overnight hydrothermal treatment at 70 °C. The MSN suspension was then centrifuged at 50,000 RCF for 15 min. The isolated pellets were resuspended, washed twice with 100% ethanol. CTAB removal was achieved by resuspending particles in 100 mL of 6 g/L ammonium nitrate in ethanol and sonicating at 40 °C for 30 min. Particles were collected by centrifugation, washed with 95% ethanol, collected by centrifugation, resuspended in 100 mL of an ethanolic HCl solution (1%) and sonicated twice for 30 min at 40 °C. Particles were washed with 90% ethanol followed by 100% ethanol, collected by centrifugation and resuspended in 40 mL of 100% ethanol.

A small batch of fluorescently labeled (Cy3) particles was prepared by dissolving CTAB (250 mg) in 150 ml of aqueous ammonium hydroxide (0.32 M) in a 250 mL beaker. The reaction was covered with parafilm (Bemis) and heated to 50 °C in a silicone oil bath for 1 h stirring at high speed (650 rpm). Cy3-NHS (3 mg) (ThermoFisher) was dissolved in 1 mL 100% ethanol by sonication followed by the addition of 2.5 μL of APTES (Sigma). The Cy3 solution was incubated at room temperature shielded from light for 1 h. A TEOS solution was prepared at 0.88 M in ethanol (3 mL) was combined with the Cy3-APTES solution and added to the CTAB solution and stirred vigorously at 50 °C for one hour, unsealed then aged overnight (~18 h) in the 50 °C oil bath without stirring. The remaining volume was then transferred to a 100 mL glass bottle and capped for an overnight hydrothermal treatment at 70 °C. The MSN suspension was then centrifuged at 50,000 RCF for 15 min. and particles were washed twice with 100% ethanol. CTAB removal was achieved by resuspending particles in 20 mL of 6 g/L ammonium nitrate in ethanol and sonicating at 40 °C for 20 min. Particles were collected by centrifugation, washed with 95% ethanol, collected by centrifugation, resuspended in 20 mL of an ethanolic HCl solution (1%) and sonicated twice for 20 min at 40 °C. Cy3-labelled particles were washed with 90% ethanol followed by 100% ethanol, collected by centrifugation and resuspended in 12 mL of 100% ethanol.

Prior to use, all MSN suspensions were passed through a 1 μm filter to remove any eventual large aggregates. Quantification was carried out by weight after desiccation of three 500 μL aliquots. Size and zeta potential were measured using a Zetasizer instrument (Malvern Instruments, Ltd). Morphology was assessed with TEM (JEOL 2010). For porosimetry measurements nitrogen sorption data was collected at 77 K with a Quantachrome AutoSorb-iQ2 sorption analyzer, after degassing the samples under vacuum at 333 K. Surface areas were determined using the Brunauer–Emmett–Teller (BET) model. Non-Local Density Functional Theory (NLDFT) was used to calculate pore size distributions and surface areas assuming the surface to be silica with cylindrical pores. Pore size distributions were also calculated according to the Barrett Joyner Halenda (BJH) method. The SEM analysis was carried out using a probe-corrected Hitachi HF5000 TEM/STEM at 200 kV in STEM mode. The STEM unit is equipped with a secondary electron (SE) detector in addition to the annular dark field (ADF), annular bright field (ABF) and bright field (BF) detectors, which allow simultaneous secondary and transmitted election imaging to obtain information from the surface (SE) and bulk (ADF and BF) of the nanomaterial.

### LC-MSN fabrication and characterization

Liposomes (5 mg) were prepared by combining 77.5 mol% 1,2-distearoyl-*sn*-glycero-3-phosphocholine (DSPC), 20 mol% cholesterol, and 2.5 mol% 1,2-distearoyl-*sn*-glycero-3-phosphoethanolamine-N-[methoxy(polyethylene glycol)-2000] (DSPE-PEG2000) (Avanti Polar Lipids) in chloroform. Lipid films were prepared by evaporating the chloroform using a rotary evaporator (Buchi Corp.), incubated under vacuum overnight, and rehydrated at a 5 mg/mL concentration in a 0.5x PBS (Gibco/Life Technologies), 4 mM MgCl_2_ solution for 30 min at 55 °C. The lipid solution was purged with nitrogen for two minutes and then dispersed with an ultrasonication probe (Branson Sonifier, Emerson US). Lipids were sonicated under nitrogen at 10–12 watts for 4 min, followed by a 2 min rest period, repeated twice. Lipids were centrifuged at 16,000 RCF for 20 min to remove any debris.

Loaded LC-MSNs were prepared by resuspending 1 mg MSNs in 10 µL water followed by overnight incubation at 4 °C in 100 µl of 1 mg/mL ML336 (Cayman Chemicals) in dimethyl sulfoxide (DMSO) (100 µL DMSO for unloaded LC-MSN groups). To form LC-MSNs, the resulting liposomes were combined with MSNs under bath sonication (30 sec) while pipetting at a 5:1 mass ratio of liposomes:nanoparticles. Particles were then centrifuged at 21,000 RCF and unbound liposomes were removed with the supernatant. This latter was saved for further loading efficiency analyses. When used immediately, resulting LC-MSNs were rinsed twice by resuspending in 1 mL PBS, centrifuging at 21,000 RCF, and removing supernatant. For storing LC-MSNs, particles were rinsed once in PBS and then resuspended in a 9 wt% sucrose solution in PBS, flash-frozen in liquid nitrogen, and then stored at −80 °C. Prior to use (e.g. cryo-EM analysis and all *in vitro* and *in vivo* studies), the particles were thawed and rinsed once in PBS. For animal studies with viral infection, LC-MSNs were prepared in 2 mg aliquots, combined, and redistributed into 1 or 1.5 mg aliquots before freezing for storage.

Dynamic light scattering (DLS) for particles hydrodynamic diameter and polydispersity index (PDI), and zeta potential measurements were obtained using a Malvern Zetasizer. For cryo-EM analysis, unloaded and loaded LC-MSNs were vitrified using a Vitrobot (Thermo Fisher Scientific) as previously described^43^. Briefly, 3 µL of a particle suspension was placed on a C-flat grid (Protochips, Inc.) with 2 µm diameter holes, blotted with filter paper, and plunged into liquid ethane for flash freezing. Frozen grids were stored under liquid nitrogen and transferred to an electron microscope JEM 2200FS (JEOL Ltd). Grids were imaged at 200 keV using DE-20 (Direct Detector Inc.) direct electron detector camera. The 2200FS microscope had a field emission electron source and an omega-type electron energy filter to remove inelastically scattered electrons from image formation. The energy selecting slit was set to 20 eV. A DE-20 camera was used in “movie” mode with frame rate of 25 frames/s. Off-line frame alignment was performed with DE_process_frames.py script provided by Direct Electron Inc. Images were collected at 40,000x indicated magnification, the pixel size on the specimen scale corresponded to 1.5 Å/pixel. Images were collected with 1.5 to 2.6 µm defocus range. Cryo-EM images were analyzed for size and lipid bilayer thickness using ImageJ. Fifty particles were analyzed from each of the ML336-loaded and unloaded LC-MSN groups.

### LC-MSN ML336 loading and release studies

The concentration of ML336 was determined by correlating sample (supernatants) spectroscopic absorption measurements at 320 nm (Nanodrop, ThermoFisher Scientific) with ML336 standard curves prepared in the 5 mg/mL liposome suspension (described above) or PBS (Fig. S2C). Loading of ML336 in LC-MSNs was calculated using the following formula: Total mass loaded = Initial mass of ML336 added − [(mass of ML336 in the supernatant after combination with the lipids) + (mass of ML336 in the supernatant of PBS wash 1) + (mass of ML336 in the supernatant of PBS wash 2)]. A dataset of six replicates highlights further how loading and release was calculated in the supplementary information (Fig. S2D). Briefly, to determine the mass of ML336 loaded, the mass of ML336 in the supernatant from each wash step (one after lipid application and two in PBS, labeled A, B, and C, respectively, in Fig. S2D) was subtracted from the total mass of ML336 loaded, 100 µg (100 µg − (A + B + C); Fig. S2D). The total loading was then reported on a per mass LC-MSN basis.

For release studies, rinsed LC-MSNs were resuspended in 1 mL of PBS and incubated at room temperature. Release of ML336 was measured by removing 100 µL aliquots from each sample, centrifuging the aliquot at 21,000 RCF, and measuring the concentration of ML336 in the supernatant at 0.25, 0.5, 0.75, 1, 2, 3, 4, 24, and 96 h timepoints. Cumulative mass released was calculated by averaging the mass observed in each sample at each timepoint. Cumulative percent released was calculated by averaging the percent released for each sample at each timepoint using the following formula: Percent released = 100%*(mass released at timepoint X/total mass loaded).

### LC-MSN internalization studies

All cells were maintained at 37 °C and 5% CO_2_. Cells were maintained in Dulbecco’s Modified Eagle Medium (DMEM, Gibco/Life Technologies; HeLas) or Minimum Essential Medium α (αMEM, Gibco/Life Technologies; Veros) supplemented with 10 vol% FBS (Hyclone), 10,000 IU/mL penicillin, and 10,000 µg/ml streptomycin (MP Biomedicals). For endocytosis inhibitor studies with LC-MSNs, HeLa cells were plated overnight in complete medium on 12-well plates for image analysis or flow cytometry. Unless otherwise indicated, all inhibitors were purchased from EMD Millipore. The following inhibitors were initially resuspended in DMSO and then diluted in complete medium to obtain the final concentrations indicated: vacuolar H^+^-ATPase inhibitor bafilomycin A (BAF)(100 nM), cationic amphiphilic compound chlorpromazine (CPZ; 6.5 μg/ml; Sigma), dynamin 2 inhibitor dynasore (DYN) (80 μM), wortmannin (Wort) (150 ng/ml), p21-activated kinase inhibitor III (IPA-3) (10 μM), and phorbol 12-myristate 13-acetate (PMA) (10 μM). Cells were incubated with inhibitor treatments for 1 h prior and during incubation with Cy3 labeled LC-MSNs (25 µg MSN/well), or the pathogens Vesicular Stomatitis virus (VSV strain Indiana 1) or Rift Valley fever virus (RVFV strain MP-12) used as specificity controls for clathrin-mediated endocytosis and caveolae-mediated endocytosis, respectively. For image analysis studies, the cells were washed at 5 h post LC-MSN addition with PBS twice and then subjected to brightfield and fluorescent microscopy. For flow cytometry experiments, cells were washed with PBS twice at 16 h post LC-MSN addition or virus infection, then prepared for analysis on a BD Accuri C6 instrument (Becton, Dickerson and Company). Flow cytometry data were analyzed by FCS Express v6 software (De Novo Software).

For confocal microscopy imaging of cellular association with LC-MSNs, HeLa cells were seeded overnight onto No. 1.5 glass cover slips in 6-well plates at a density of 0.75 × 10^5^ cells per well. Fluorescent (Cy3) LC-MSNs were added at 25 µg MSN/well and incubated for 45 min or 20 h. After incubation, cells were washed with PBS, fixed with 4% paraformaldehyde in PBS for 15 min with prewarmed solutions followed by overnight storage at 4 °C, washed twice with PBS, and made permeable with 0.1% Triton-X in PBS for 15 min. Cells were then blocked with 1% BSA in PBS for 20 min and then labeled with 5 units/1 ml Alexa Fluor 647 phalloidin (ThermoFisher) and Alexa Fluor 488 anti-α-tubulin antibody (Invitrogen) in blocking buffer for 1 h. After washing with PBS, slides were mounted using Prolong Gold with DAPI (ThermoFisher). Confocal images were acquired with a 63X/1.4NA oil objective in sequential scanning mode using a Leica TCS SP8 confocal microscope. Three dimensional cell images were isosurface rendered using the Leica Application Suite Advanced Fluorescence 3D analysis software.

### *In vitro* viral inhibition

The TC-83 virus was obtained through the NIH Biodefense and Emerging Infections Research Resources Repository, NIAID, NIH (NR-63), and was propagated in Vero cells. Cells were infected at a multiplicities of infection (MOI) of 0.1 and cultured for two days. Supernatant was collected and the concentration of plaque forming units (PFUs) was determined using a standard plaque assay with an agarose overlay (1:1 2X Modified Eagle Medium (Gibco/Life Technologies; 8 vol% FBS, 10,000 IU/mL penicillin, and 10,000 µg/ml streptomycin):1.5 wt% agarose (Invitrogen)). Cells were fixed and stained with an ethanol-based crystal violet solution (0.14 wt% crystal violet (Sigma-Aldrich), 21 vol% ethanol) and plaques were counted manually to determine PFU/mL.

HeLa cells at 80–90% confluency in 12 well plates were pretreated with 25 µg LC-MSNs in 100 µL Opti-MEM Reduced Serum Media (Gibco/Life Technologies) for 1 h prior to infection with TC-83 at 0.1 MOI for 30–60 min. Virus was then removed, cells were rinsed three times in PBS, and treatments were added back for the remainder of the time course in 1 mL DMEM. Supernatants were taken at 24, 48, and 72 h. Phase images were taken at the same timepoints using an inverted microscope (Olympus- IX70).

For experiments with pre-released LC-MSNs, LC-MSNs were incubated in Opti-MEM at a concentration of 2.5 µg/mL for 4 h at room temperature. LC-MSNs were then centrifuged at 20,000 RCF and supernatant was collected. Particles were resuspended at 2.5 µg/mL in Opti-MEM and immediately added to cell cultures as described above. The supernatant of 25 µg of particles (100 µL) was also immediately added to cells.

The concentration of PFUs in supernatants was determined using a standard plaque assay on Vero cells in 12 well plates as described above. Serial dilutions of supernatants were prepared in αMEM and used to infect cells for 30–60 min. Due to the minimum concentration of virions in supernatants required for detection in plaque assays, the limit of detection (indicated on each graph) was 100 PFU/mL.

### *In vivo* viral inhibition

All animal work was conducted in accordance with protocols approved by the Lawrence Livermore National Laboratory Institution Animal Care and Use Committee. For safety studies, five 6–8 week old C3H/HeN mice were injected intraperitoneal (IP) with 1 mg LC-MSNs in 200 µL PBS and 3 mice were injected with 200 µL PBS only. Mice were monitored for 15 days and weighed on days 3, 6, 9, and 15. At day 15, animals were euthanized and lungs, livers, spleens, kidneys, and brains were dissected from three animals in each group.

An established C3H/HeN murine model of VEEV infection was used to assess the ability of ML336-loaded LC-MSNs to inhibit viral infection^8^. In the first study, animals were divided into four groups of ten mice each: ML336-loaded LC-MSNs, unloaded LC-MSNs, free drug, and PBS groups. For ML336-loaded and unloaded LC-MSNs groups, 1 mg of LC-MSNs was mixed with 200 µL 1 wt% sodium carboxymethylcellulose (Sigma-Aldrich) in PBS. For free drug groups, 20 µL of 1 mg/mL ML336 in DMSO was mixed into 200 µL of 1 wt% sodium carboxymethylcellulose in PBS, resulting in 20 µg ML336 per injection, similar to what is loaded in 1 mg ML336-loaded LC-MSNs. Mice were injected twice a day IP for 4 days. Four hours after the first injection on day 1, the mice were infected with an intranasal instillation of 50 μl of TC-83 containing a total of 10^8^ PFU. Mice were monitored for weight and clinical signs of disease each day post-infection and assigned a clinical score from 0–4 (mild to severe). 0 = Bright, alert, responsive and active. Animals exhibit normal grooming and social behavior. No loss of appetite. 1 = Mild clinical signs of infection such as coat ruffling and loss of appetite. 2 = Pronounced decrease in activity and responsiveness to stimulation. Ruffled coat and rapid, shallow breathing. Obvious neurological impairment such as trouble ambulating and hunching. 3 = Moribund. Eyes closed completely, labored breathing. No activity and unresponsive to tail tug. Or, animals that have lost > 25% of their body weight. Mice that met this criterion were euthanized. 4 = Found dead.

The second animal study was conducted as described above with the following changes. LC-MSN masses for both loaded and unloaded LC-MSN groups were increased to 1.5 mg. For free drug groups,15 µL of 2 mg/mL ML336 in DMSO was mixed into 185 µL of 1 wt% sodium carboxymethylcellulose in PBS, resulting in 30 µg ML336 per injection, similar to what is loaded in 1.5 mg ML336-loaded LC-MSNs. On day 5, five mice from each group were euthanized and dissected for brain, spleen, kidney, liver, and serum. Organs were homogenized using disposable tissue grinders and tissue lysate was assessed for viral load using a standard plaque assay as described above.

### Statistical Analysis

All results are depicted as mean ± standard deviation. Analysis of Variance (ANOVA) was used to identify significant factors and interactions, then Tukey’s post hoc test (significance level p ≤ 0.05) was used to generate pairwise comparisons between means of individual sample groups and determine statistical significance (GraphPad Prism 7).

## Electronic supplementary material

Supporting Information
