# Supplementary material for: Lipid-Coated Mesoporous Silica Nanoparticles for the Delivery of the ML336 Antiviral to Inhibit Encephalitic Alphavirus Infection
Source: Sci Rep. 2018 Sep 18;8:13990. doi: 10.1038/s41598-018-32033-w (PMC6143628; doi:10.1038/s41598-018-32033-w)
Supplement: Supplementary file 1 — Supporting Information [file 41598_2018_32033_MOESM1_ESM.pdf]

Supporting Information (SI)

Lipid-Coated Mesoporous Silica Nanoparticles for the Delivery of the ML336 Antiviral to Inhibit Encephalitic Alphavirus Infection

Annette E. LaBauve[1], Torri E. Rinker[1], Achraf Noureddine[2,3,4], Rita E. Serda[2,3,4], Jane Y. Howe[5], Michael B. Sherman[6], Amy Rasley[7], C. Jeffery Brinker[2,3,4], Darryl Y. Sasaki[1], Oscar A. Negrete[1]

[1]Department of Biotechnology and Bioengineering, Sandia National Laboratories, Livermore, CA. [2]Advanced Materials Laboratory, Sandia National Laboratories, Albuquerque, NM. [3]Chemical and Biological Engineering, University of New Mexico, Albuquerque, NM. [4]Center for Micro-Engineered Materials, Advanced Materials Laboratory, Albuquerque, NM. [5]Hitachi High Technologies America, Inc., Clarksburg, MD. [6]Sealy Center for Structural Biology & Molecular Biophysics, University of Texas Medical Branch, Galveston, TX. [7]Biosciences and Biotechnology Division, Lawrence Livermore National Laboratory, Livermore, CA.

Correspondence: Oscar A. Negrete: onegret@sandia.gov

Supporting information contains:

1. Supplementary figure: Fig. S1
2. Supplementary figure: Fig. S2
3. Supplementary figure: Fig. S3
4. Supplementary figure: Fig. S4
5. Supplementary figure: Fig. S5
6. Supplementary figure: Fig. S6
7. Supplementary figure: Fig. S7
8. Supplementary figure: Fig. S8
9. Supplementary figure: Fig. S9

Supplementary Figures

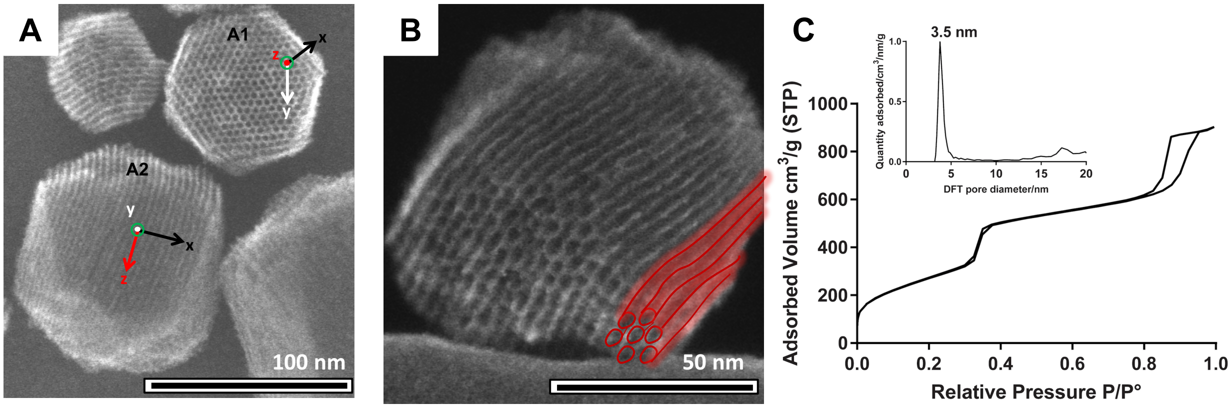

Fig. S1. Ultrastructure and pore analysis of mesoporous silica nanoparticles. (A and B) SEM images showing the hexagonal porous structure of MSNs with different projections. (A)Hexagonal structure is highlighted by the honey comb-like arrangement (A1) and tubular channels (A2). Axis (z) is parallel to the pores plan. (B) Tilted image showing tubular channels in a hexagonal arrangement ending by cargo-accessible openings (pore mouth). (C) N2 adsorption-desorption isotherm and pore size distribution (inset) for hexagonal small pore MSN.

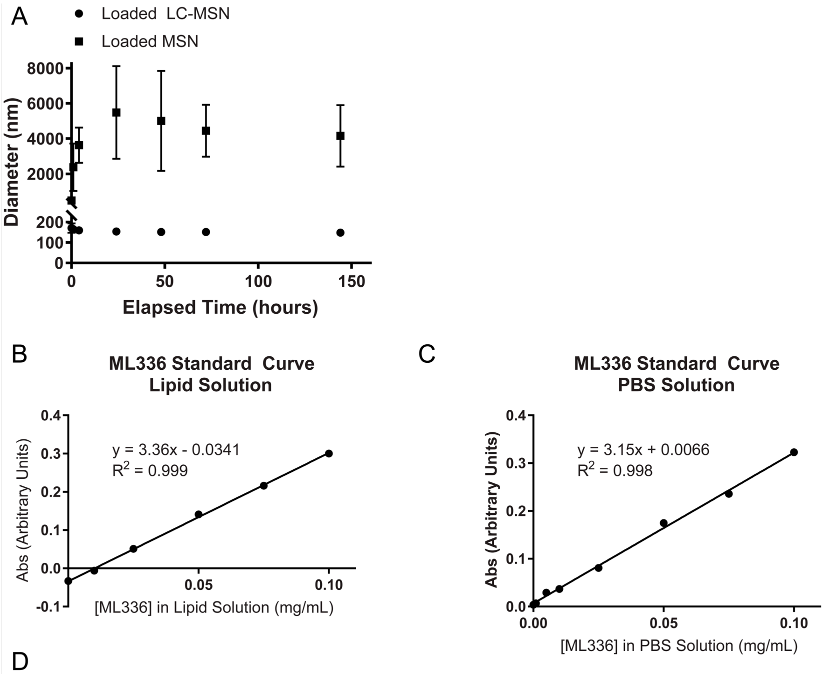

D

	Mass ML336 (ug)/mg LC-MSN in:			Total Loaded mass
	A: Supernatant after combination with lipids	B: Supernatant after PBS Wash 1	C: Supernatant after PBS Wash 2	ML336 loaded (ug) = 100 ug - (A+B+C)
**Sample 1**	70.57	5.51	3.29	20.64
**Sample 2**	69.08	7.73	4.56	18.64
**Sample 3**	72.36	8.04	2.34	17.26
**Sample 4**	72.65	8.04	2.97	16.33
**Sample 5**	69.98	5.82	2.97	21.23
**Sample 6**	63.72	7.09	3.29	25.90
			Average:	20.00
			Stdev:	3.45

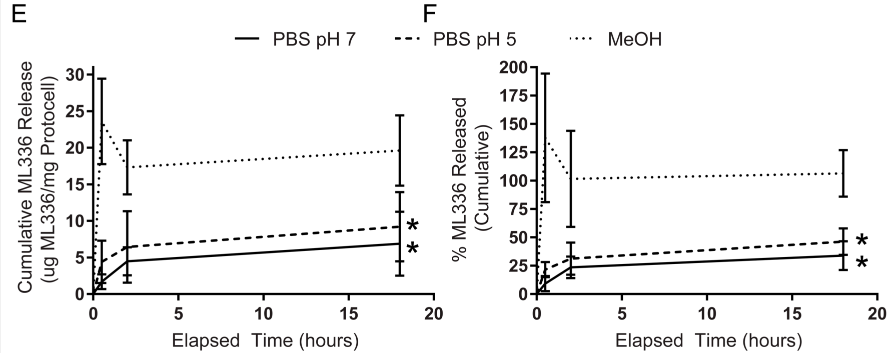

Fig. S2. (A) DLS measurements of ML336 loaded MSNs and loaded LC-MSNs over the course of a week. (B) ML336 loading and release was determined by comparing sample absorbance values at 320 nm to a standard curve in either lipid solution or (C) PBS solution. (D) ML336 loading was calculated as shown in the above table, using the following formula: Total mass loaded = Initial mass of ML336 added – [(mass of ML336 in the supernatant after combination with the lipids) + (mass of ML336 in the supernatant of PBS wash 1) + (mass of ML336 in the supernatant of PBS wash 2)] (E) Cumulative and (F) percent release (normalized to total ML336 loaded) of ML336 from LC-MSNs in PBS pH 7, PBS pH 5, and methanol (MeOH). Data represent mean ± standard deviation (*= significantly different MeOH group at 18 hours; n=5)

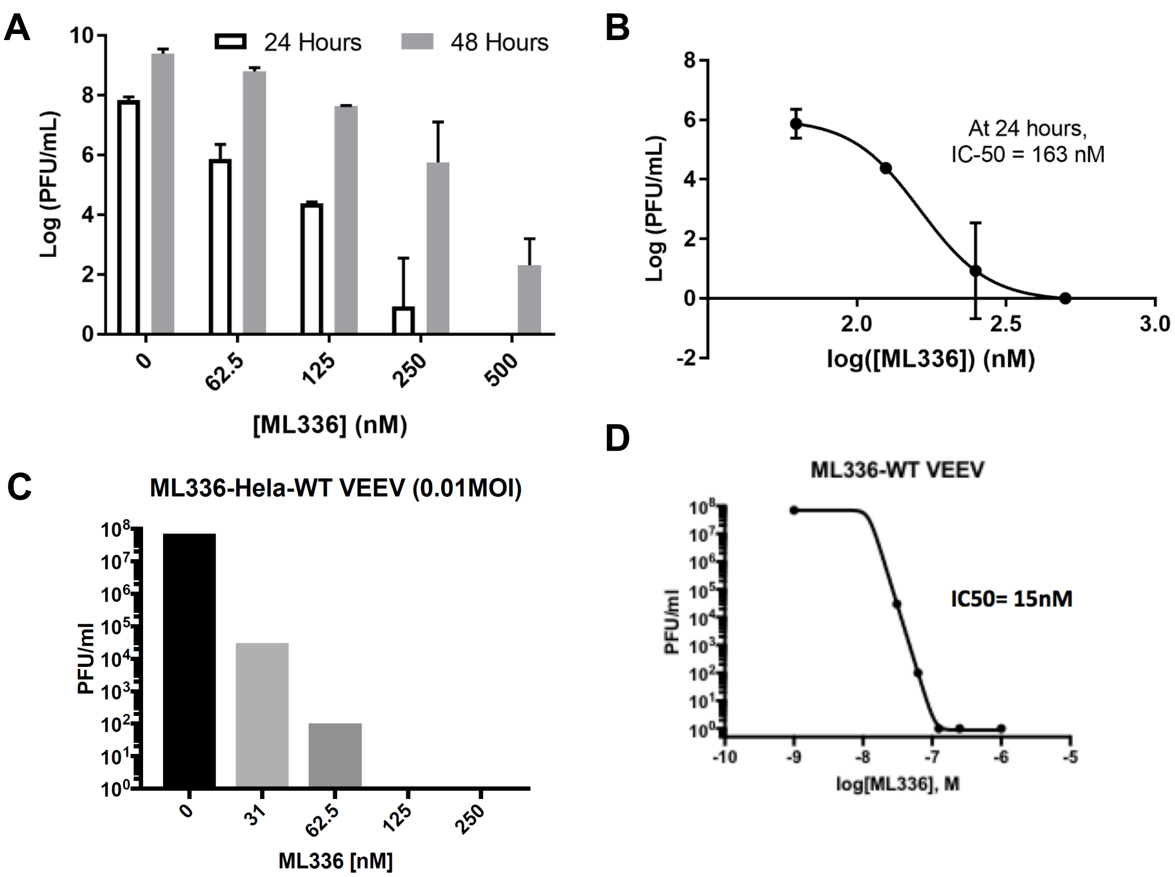

Fig. S3. ML336 inhibits TC-83 and VEEV (ZPC738 virulent strain) in a dose-dependent manner. (A) TC-83 viral inhibition in HeLa cells at 24 and 48 hours with increasing concentrations of ML336 (n=3). (B) IC-50 of ML336 is 163 nm for HeLa cells at 24 hours of TC-83 infection. (C) VEEV viral inhibition in HeLa cells at 24 hours with increasing concentrations of ML336. (D) IC-50 of ML336 is 15 nm for HeLa cells at 24 hours of VEEV infection (n=1).

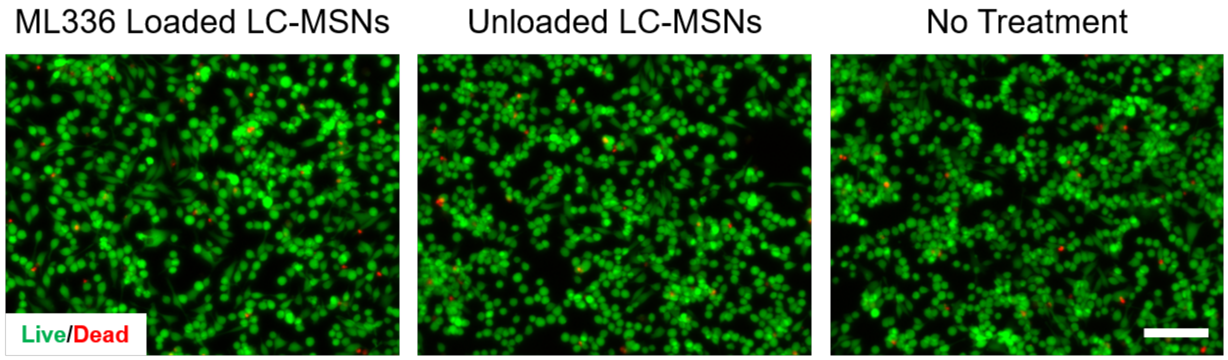

Fig. S4. ML336 loaded LC-MSNs do not visibly affect cell viability. LIVE (green)/DEAD (red) staining on cells treated with ML336 loaded LC-MSNs, unloaded LC-MSNs, or nothing for 48 hours (scale bar = 50 µm; n=3)

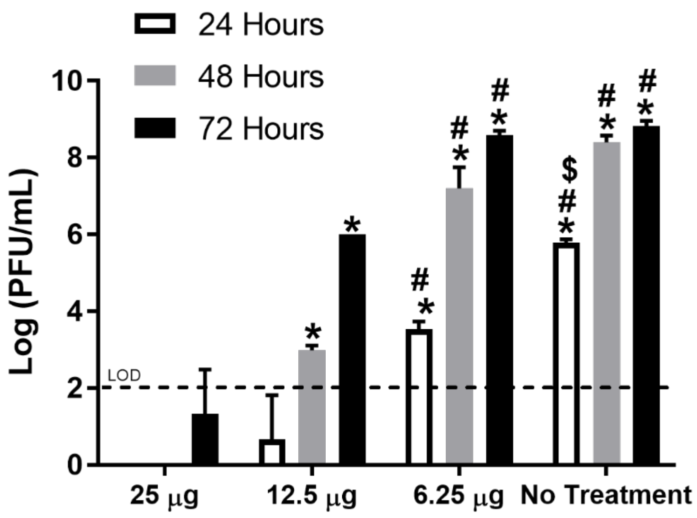

Fig. S5. ML336 loaded LC-MSNs inhibit virus in a dose-dependent manner (*= significantly different than 25 µg group at same timepoint, # = significantly different than 12.5 µg at the same timepoint, $ = significantly different than 6.25 µg group at the same timepoint; $p<0.05$; data is depicted as mean ± standard deviation; n=3).

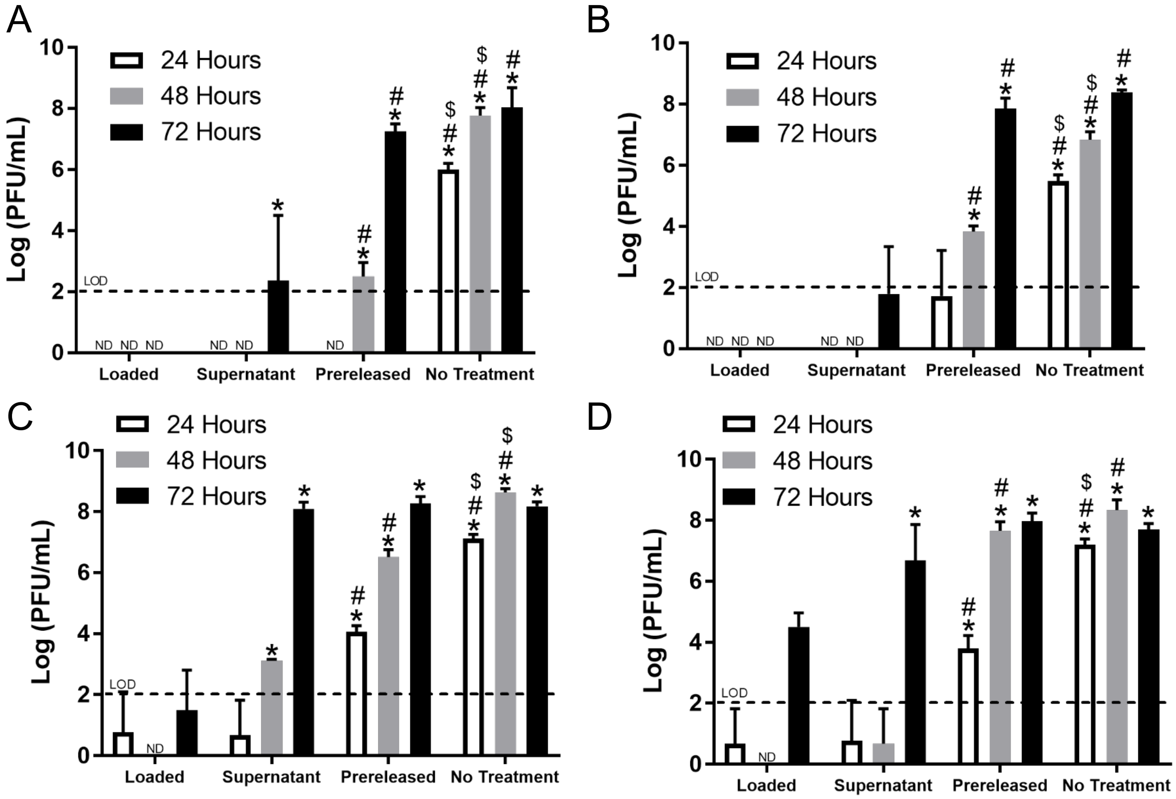

Fig. S6. Repeatability studies for LC-MSN viral inhibition *in vitro*. PFU/mL for loaded, supernatant, pre-released, and untreated groups for batch 1 (A and C) and batch 2 (B and D) in study 1 (A and B) and study 2 (C and D). Note that (A) is also depicted in Figure 3C. (*= significantly different than loaded group at same timepoint, # = significantly different than supernatant group at the same timepoint, $ = significantly different than pre-released group at the same timepoint; $p<0.05$; data is depicted as mean ± standard deviation; n=3 technical replicates and 5 biological replicates).

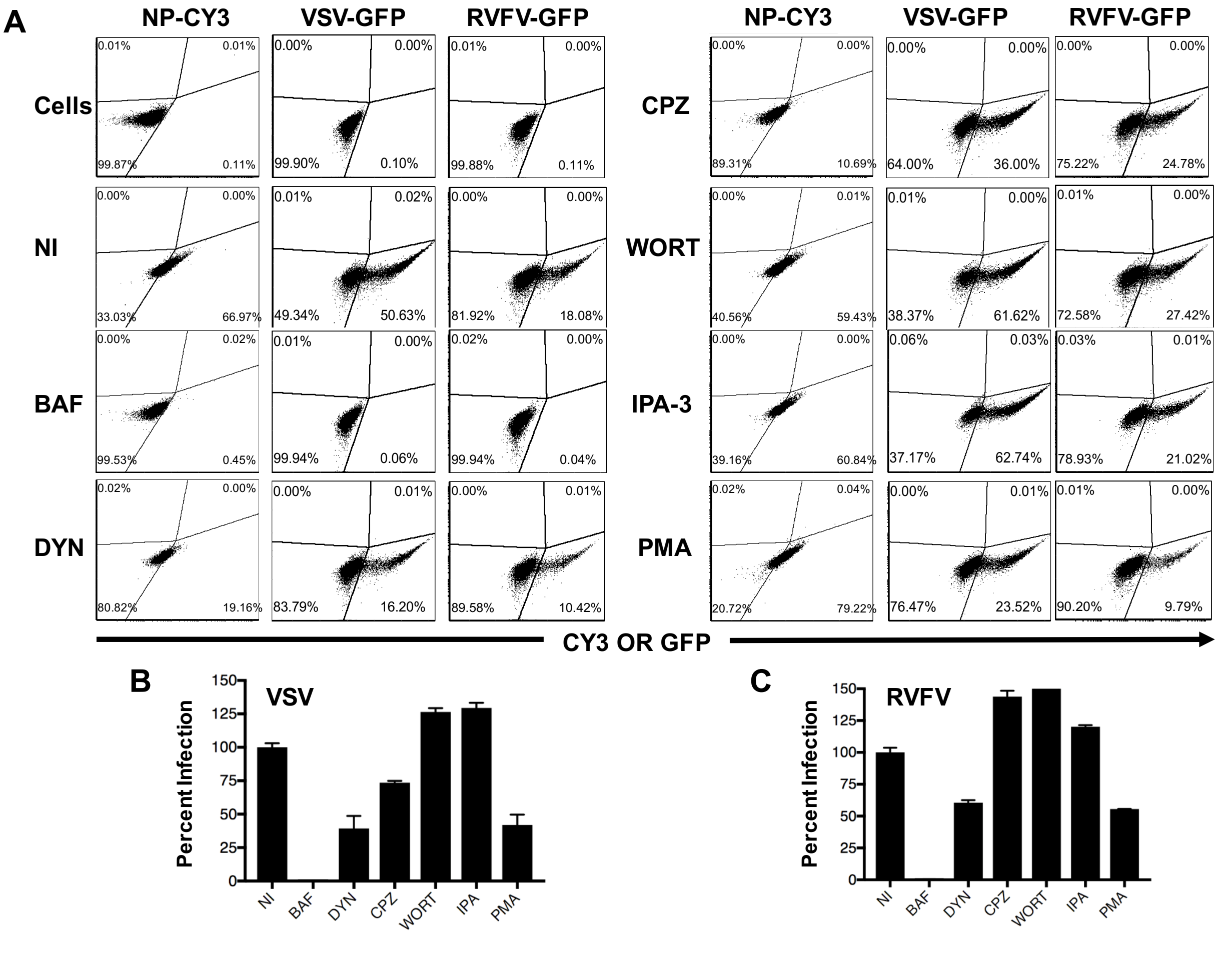

Fig. S7. Inhibitors of clathrin-mediated endocytosis reduce both LC-MSN uptake and VSV infection, while inhibitors of caveolae-mediated endocytosis block RVFV and some VSV infection, but not LC-MSN cellular internalization. (A) HeLa cells were incubated with endocytosis inhibitor treatments for 1h prior and during incubation with Cy3 labeled LC-MSNs (NP-Cy3), Vesicular Stomatitis virus (VSV) or Rift Valley fever virus (RVFV strain MP-12). At 16 h post nanoparticle or virus addition, cells were washed with PBS twice and prepared for flow cytometry analysis. Representative flow data are shown. No inhibitor (NI) control values were used to normalize the treatment conditions as percent infection for (B) VSV or (C) RVFV (n=2). Overall, these treatment conditions specifically distinguish between common endocytic pathways.

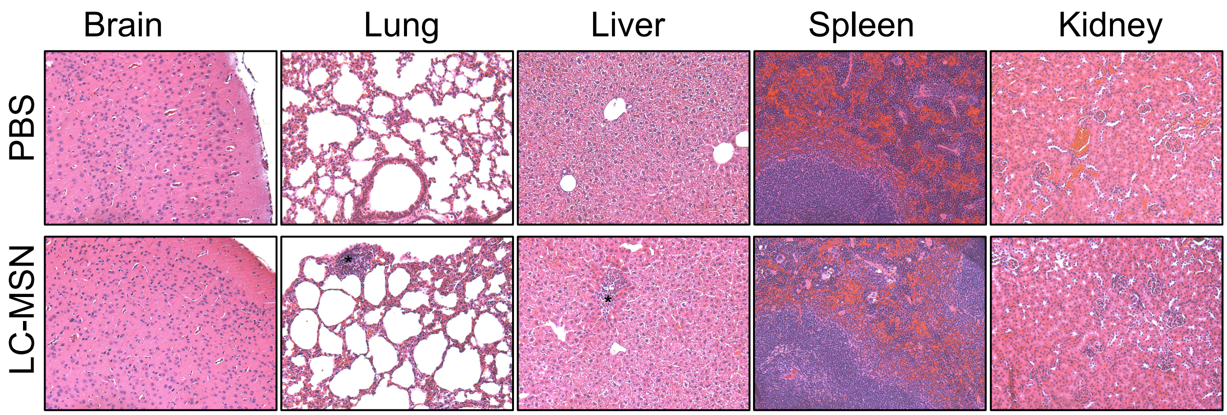

Fig. S8. Histological analysis of LC-MSN dosed C3H/HeN mice. Mouse tissues were dissected and formalin-fixed on day 15 post-treatment with a vehicle control (PBS) or LC-MSNs at 0.11g LC-MSNs/kg/day for four days**.** Histological specimens were prepared through paraffin embedding and sectioning, followed by hematoxylin and eosin staining. Three animals per group were analyzed and representative images are shown. In the brain, the outer cortex is shown and displayed no obvious differences between the LC-MSN dosed and control groups. Similarly, the spleen and kidney sections exhibited normal morphology without signs of toxicity. In some samples, granulomas that contained collections of macrophages embedded in the lung and liver (indicated by asterisks) were identified and indicative of very mild symptoms.

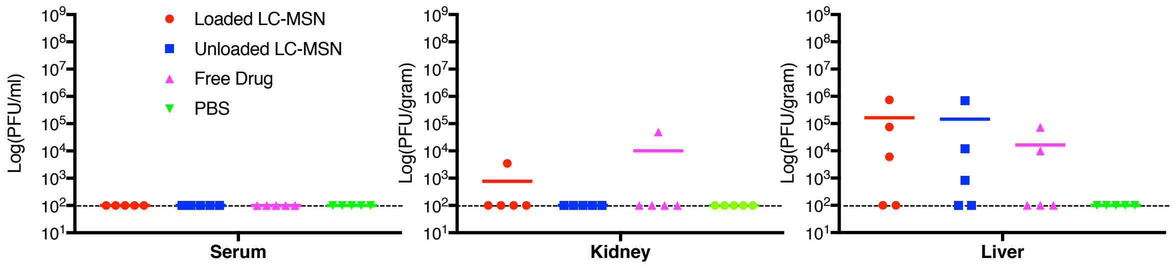

Fig. S9. Viral titer in tissues of TC-83 infected mice. The viral loads in serum (left), kidney (middle), and liver (right) at day 4 post-infection via intranasal challenge of C3H/HeN mice with VEEV strain TC-83 were measured by standard plaque assays normalized to volume (ml) or organs mass (gram). Viral loads from four treatment conditions are shown as follows, ML-336 loaded LC-MSN (red), unloaded LC-MSN (blue), free ML-336 (purple), and vehicle only (PBS) (green) with mean from 5 samples per condition. The limit of detection (LOD) is 100PFU and samples at or below this threshold are all listed at LOD.
